# Self-catalytic crystal growth, formation mechanism, and optical properties of indium tin oxide nanostructures

**DOI:** 10.1186/1556-276X-8-358

**Published:** 2013-08-22

**Authors:** Yuan-Chang Liang, Hua Zhong

**Affiliations:** 1 Institute of Materials Engineering, National Taiwan Ocean University, Keelung 20224, Taiwan

**Keywords:** In-Sn-O nanostructures, Morphology, Doping, Crystal growth, Optical properties

## Abstract

In-Sn-O nanostructures with rectangular cross-sectional rod-like, sword-like, and bowling pin-like morphologies were successfully synthesized through self-catalytic growth. Mixed metallic In and Sn powders were used as source materials, and no catalyst layer was pre-coated on the substrates. The distance between the substrate and the source materials affected the size of the Sn-rich alloy particles during crystal growth in a quartz tube. This caused In-Sn-O nanostructures with various morphologies to form. An X-ray photoelectron spectroscope and a transmittance electron microscope with an energy-dispersive X-ray spectrometer were used to investigate the elemental binding states and compositions of the as-synthesized nanostructures. The Sn doping and oxygen vacancies in the In_2_O_3_ crystals corresponded to the blue-green and yellow-orange emission bands of the nanostructures, respectively.

## Background

Oxide materials are promising constituents for various scientific applications because of their versatile physical properties [1]. Oxide materials in low-dimensional forms are particularly demanded for manufacturing small devices. One-dimensional (1D) metal-oxide nanostructures with a high aspect ratio and good crystallinity are promising as building blocks for functional device architecture. Indium oxide (In_2_O_3_) is a wide bandgap semiconductor and has been used in various optoelectronic and electronic devices [2,3]. For practical applications, In_2_O_3_ semiconductors are usually doped with other elements to increase their functionalities [2,4-6].

Recently, Sn-doped In_2_O_3_ has attracted a considerable amount of attention because of its superior transparency in the visible spectral region and low electrical resistivity. Sn-doped In_2_O_3_ is the transparent conducting oxide most widely used in scientific and industrial applications. Sn-doped In_2_O_3_ can be integrated into solar cells, smart windows, photocurrent generators, displays, and light-emitting diodes [7,8]. However, most studies on Sn-doped In_2_O_3_ have mainly focused on its thin-film structure because of the numerous applications of this material in optoelectronic and electronic devices [9,10]. By contrast, there are few works on Sn-doped In_2_O_3_ regarding its 1D structure. Recently, comprehensive investigations on the 1D nanostructures of In_2_O_3_ have been conducted. In_2_O_3_ 1D nanostructures have been synthesized using several chemical and physical methodologies [11,12]. Thermal evaporation is the simplest method used to synthesize In_2_O_3_ nanostructures with a large density and high crystalline quality [13]. The source materials used to grow 1D In_2_O_3_ nanostructures through thermal evaporation include metallic In powder and ceramic In_2_O_3_ powders mixed with carbon powders. Generally, a high growth temperature is required to obtain In_2_O_3_ nanostructures when using ceramic powders as the source material. In addition to the source materials, the evaporative synthesis of these nanostructures can be further classified depending on whether or not a metallic catalyst is used during crystal growth. For optoelectronic nanodevice applications, In_2_O_3_ nanostructures are doped with trace Sn to enhance their optical and electrical characteristics [14,15]. Sn-doped In_2_O_3_ nanostructures have several superior properties including a high metallic conductivity, excellent oxidation resistance, and good thermal stability. However, recent works on Sn-doped In_2_O_3_ nanostructures with various morphologies have been limited to synthesis using mixed ceramic powders composed of various elements, such as In_2_O_3_ with SnO_2_ or InN with SnO_2_, on the substrates, with or without an Au catalyst layer [14,16]. Some Sn-doped In_2_O_3_ nanostructures were synthesized using mixed metallic In and Sn powders on Au catalyst-coated substrates [17]. In this study, Sn-doped In_2_O_3_ nanostructures with various morphologies were synthesized using mixed In and Sn powders. No metal catalyst was used to grow the nanostructures. This paper presents the detailed investigation of nanostructures that were produced through self-catalytic growth and reports the related microstructures and self-catalytic growth mechanisms of the In-Sn-O nanostructures.

## Methods

The synthesis of In-Sn-O nanostructures was performed in a horizontal quartz tube furnace. SiO_2_/Si (100) and sapphire (0001) are used as substrates. Metallic In and Sn powders were used as the solid precursor. Sn atomic percentage in the source powder is approximately 12%. The mixed powders were placed on an alumina boat and positioned at the center of a horizontal quartz tube furnace. Substrates were loaded on separate alumina boats in the source downstream at different distances (15, 20, and 21 cm apart from the source materials) respectively. The furnace tube was then heated to 800°C for source materials, and the corresponding substrate temperature ranges from 400°C to 500°C. During the growth, the pressure in the reaction tube was kept at about 1 Torr with a constant gas flow rate of 100 sccm Ar. The growth duration of the nanostructures was 1 h. After the system had cooled down to room temperature under a 20 Torr of Ar gas atmosphere, a layer of white product was found deposited on the substrates.

The crystal structure of the samples was investigated by X-ray diffraction (XRD) with Cu Kα radiation. X-ray photoelectron spectroscope (XPS) analysis was performed to determine the chemical binding states of the constituent elements of the In-Sn-O nanostructures. The detailed microstructure of the as-synthesized samples was characterized by scanning electron microscopy (SEM) and high-resolution transmission electron microscopy (HRTEM). The composition analysis was performed using energy-dispersive X-ray spectrometer (EDS) attached to the TEM. The room temperature-dependent photoluminescence (PL) spectra are obtained using the 325-nm line of a He-Cd laser.

## Results and discussion

**Figure 1:**
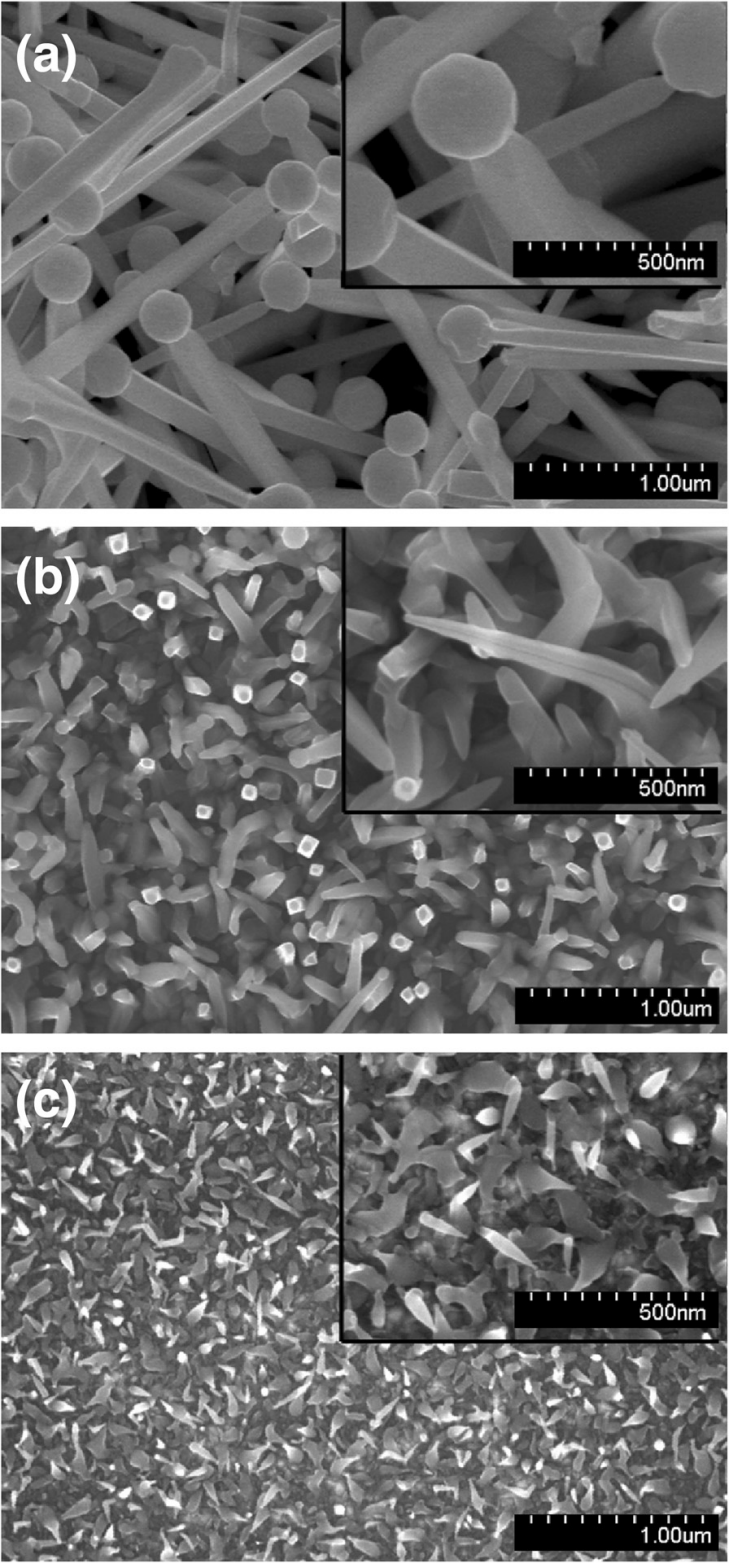
SEM images of In-Sn-O nanostructures: (a) sample 1, (b) sample 2, and (c) sample 3.

**Figure 2:**
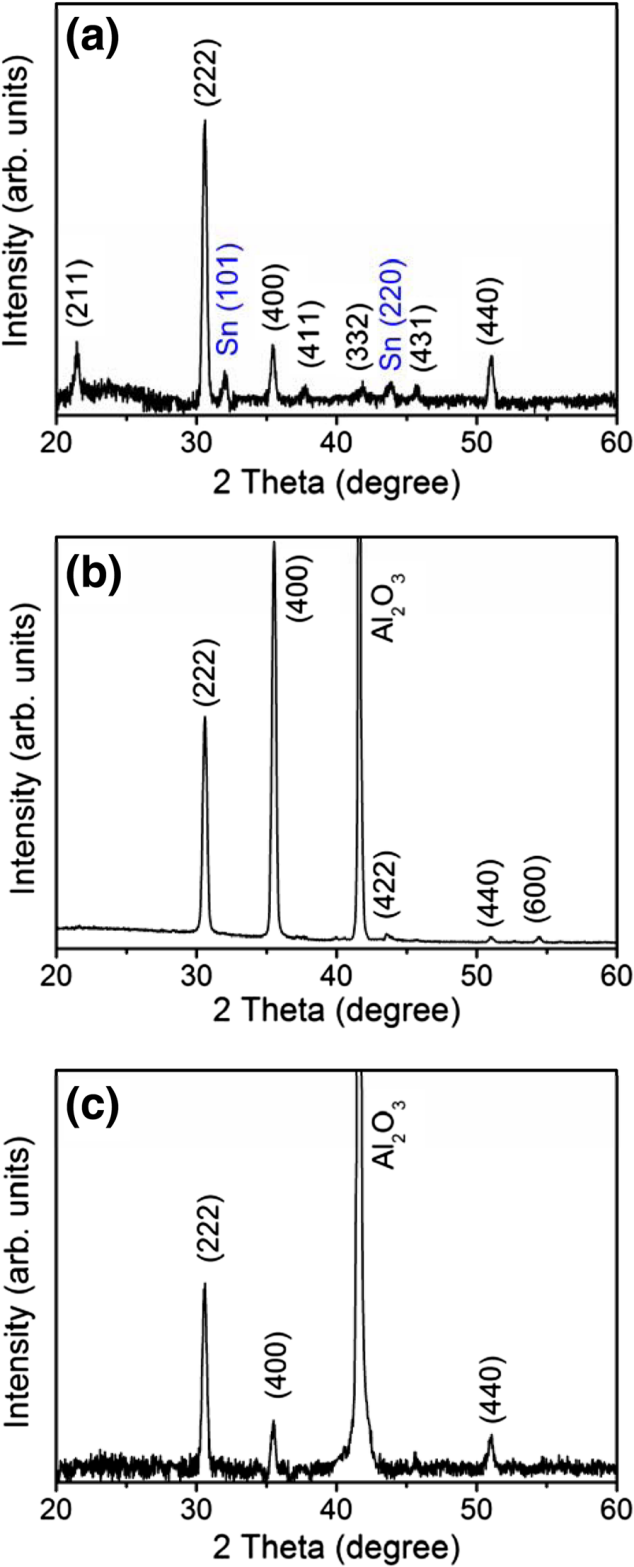
XRD patterns of In-Sn-O nanostructures: (a) sample 1, (b) sample 2, and (c) sample 3.

Figure 1 shows the SEM images of the In-Sn-O nanostructures with various morphologies, which uniformly covered the substrates. Figure 1a shows that the In-Sn-O nanostructures (sample 1) exhibited a rectangular cross-sectional stem ending in a spherical particle. The diameter of the particle was larger than the width of the stem. The width of the stems was between 100 and 200 nm. Many sword-like In-Sn-O nanostructures were observed (sample 2, Figure 1b). The morphology of sample 3 (Figure 1c) was different from that displayed in Figure 1b, showing a considerable decrease in the radius of the stem at the ending point compared with that in Figure 1b. Bowling pin-like nanostructures are the main morphological structures shown in Figure 1c. The diameter of the bottom part of stem of the nanostructures was between 40 and 80 nm. The nanostructures in Figure 1b,c also had particles at the tip. Figure 2 shows the corresponding XRD patterns of the various In-Sn-O nanostructure samples shown in Figure 1. The XRD results showed several Bragg reflections that corresponded to the cubic bixbyite of the In_2_O_3_-based phase. Several small Bragg reflections from metallic Sn appear in Figure 2a, but not in Figures 2b,c, suggesting that a high degree of metallic Sn might have been present in sample 1.

**Figure 3:**
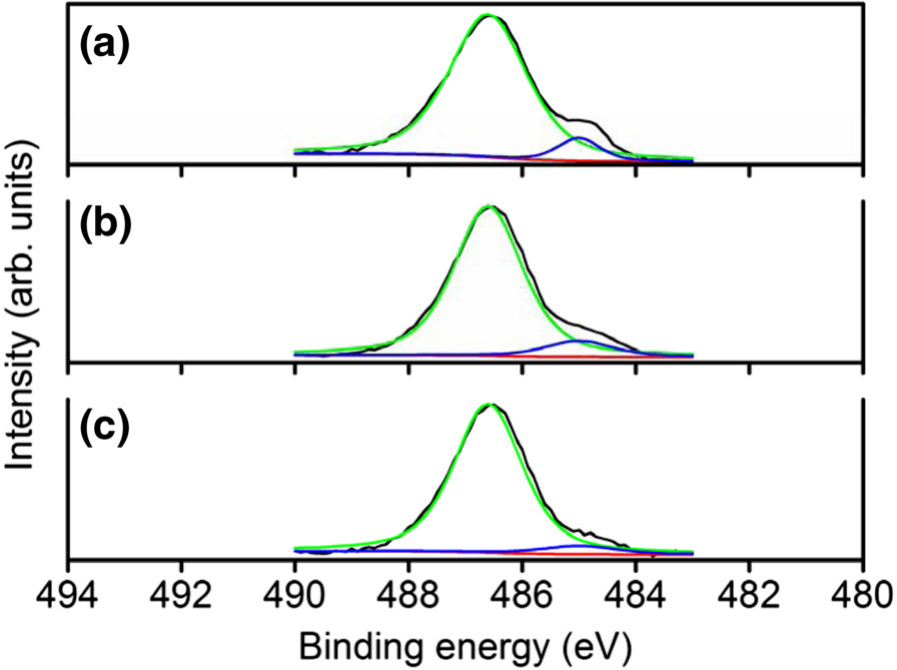
**XPS narrow scans of Sn 3*d***_**5/2 **_**core-level In-Sn-O nanostructures. (a)** Sample 1, **(b)** sample 2, and **(c)** sample 3.

**Figure 4:**
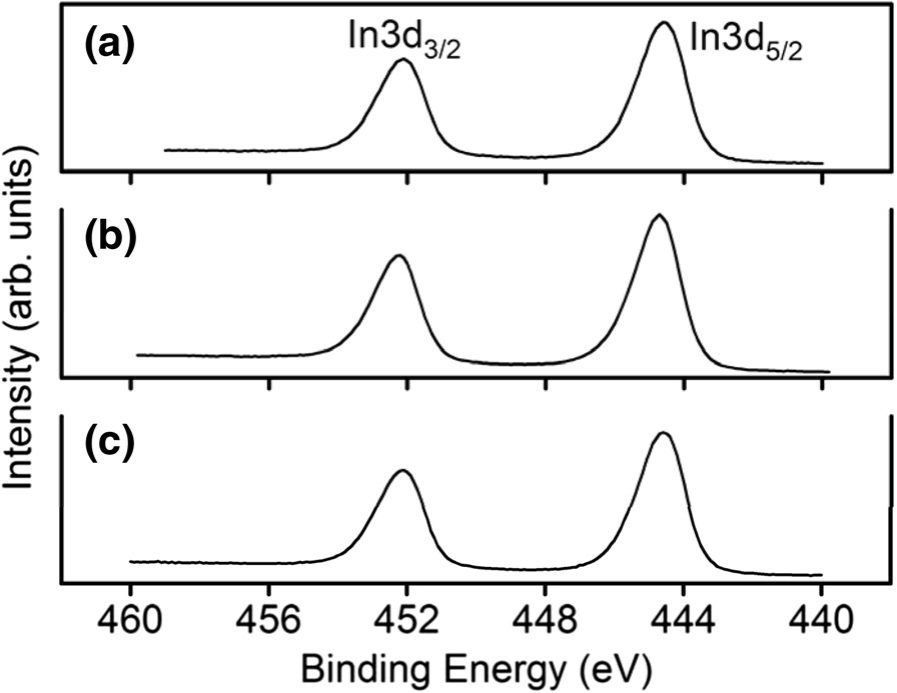
**XPS narrow scans of In 3*d *core-level doublet of In-Sn-O nanostructures. (a)** Sample 1, **(b)** sample 2, and **(c)** sample 3.

**Figure 5:**
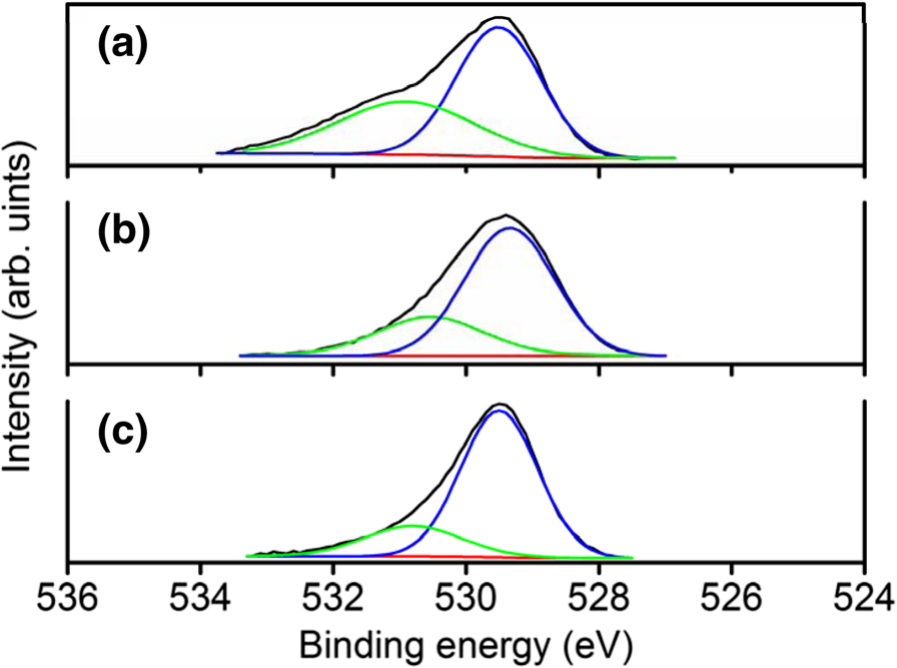
**XPS narrow scans of O 1** ***s *core level of In-Sn-O nanostructures. (a)** Sample 1, **(b)** sample 2, and **(c)** sample 3.

The Sn atomic percentages and chemical binding states of the constitutive elements of the samples were characterized using the narrow scan XPS spectra. The Sn atomic percentages of samples 1, 2, and 3 were 6.9%, 3.8%, and 3.4%, respectively. Sample 1 had a relatively large Sn content. The XPS spectra of Sn 3*d*_5/2_ showed an asymmetric curve. The detailed Gaussian-resolved results show that the two components were centered on 486.5 and 485.0 eV (Figure 3a,b,c). The relatively high binding energy component (Sn_I_) was ascribed to a Sn^4+^ valence state and that with a low binding energy (Sn_II_) was associated with metallic Sn [18,19]. The intensity ratio of Sn_II_/(Sn_I_ + Sn_II_) increased as the total Sn atomic percentages of the samples increased. Differences in morphology, particularly the dimension of the tip particles and the density of the nanostructures, might account for the various contents of metallic Sn in the samples. The composition and structure of the tip particles are identified in the following sections using TEM-EDS measurements. Figure 4a,b,c shows that the binding energies of In 3*d*_5/2_ were centered on 444.6 to 444.7 eV; these energies were associated with the In^3+^ bonding state from In_2_O_3_[20,21]. No small shoulder was observed at the lower binding energy side of the In 3*d* peaks, indicating that no In-In bonds existed in the In-Sn-O nanostructures [20]. Figure 5a,b, c shows the asymmetric O 1 *s* peaks of the samples. Two Gaussian-resolved peaks were centered on approximately 529.5 and 530.8 eV. The lower binding energy component (O_I_) was associated with oxygen in the oxide crystal, whereas the higher binding energy component (O_II_) represented the oxygen ions in the oxygen-deficient regions. Oxygen vacancies usually form in oxide nanostructures manufactured using thermal evaporation in an oxygen-deficient environment [22]. The oxygen vacancy content in the crystalline In-Sn-O nanostructures was defined as an intensity ratio: O_II_/(O_I_ + O_II_). The ratios for samples 1, 2, and 3 were 0.39, 0.28, and 0.21, respectively. Among the various nanostructures, those with a rectangular cross-sectional stem exhibited a high degree of oxygen vacancy defects.

**Figure 6:**
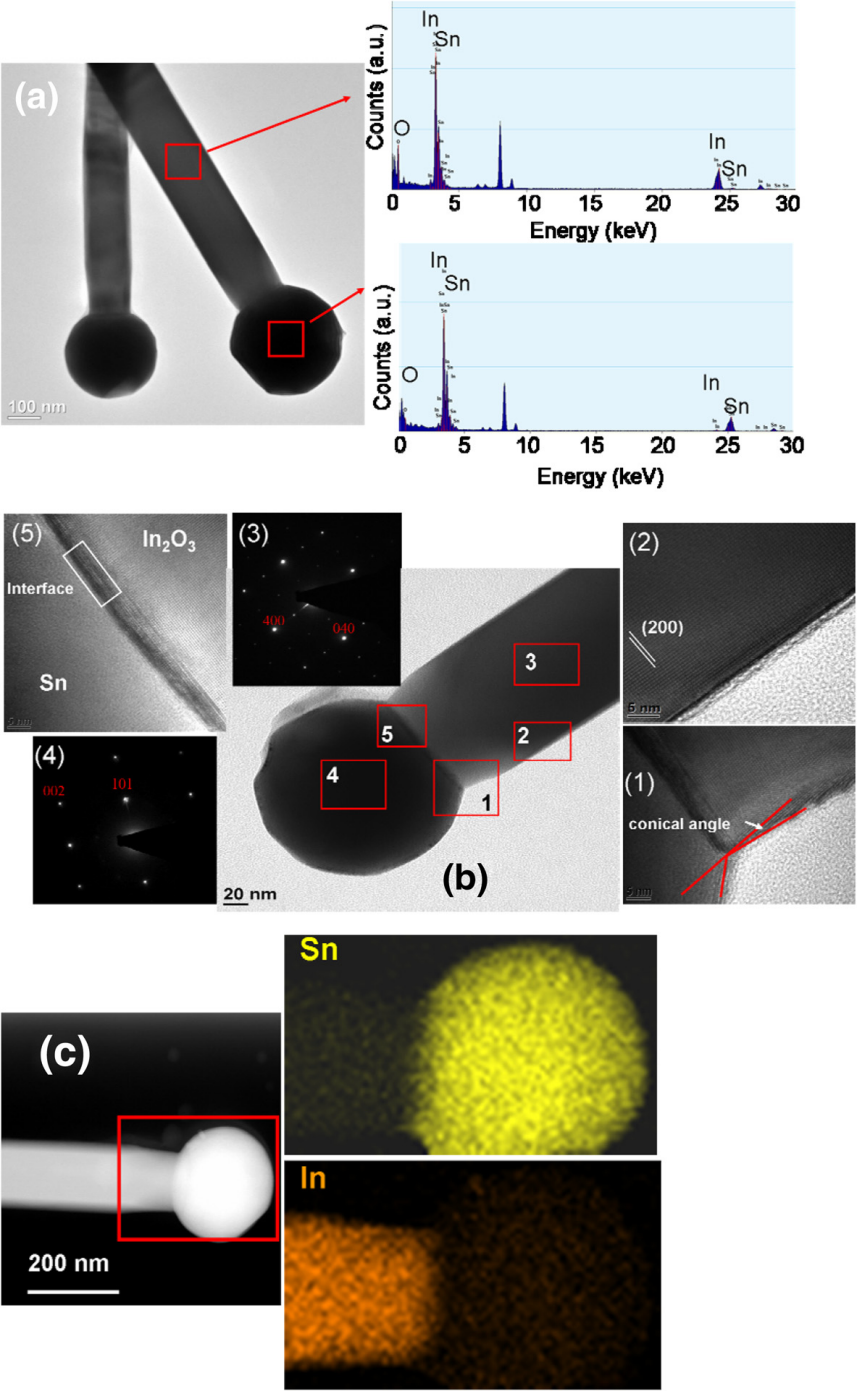
**TEM, HRTEM, and elemental mapping images of the rod-like nanostructures. (a)** Low-magnification TEM image of several In-Sn-O nanostructures. The EDS spectra taken from the stem and particle were also displayed. **(b)** HRTEM images taken from the different regions of the individual nanostructure and the selected area electron diffraction patterns from the stem and particle. **(c)** The In and Sn elemental mapping images taken from the red square region of the nanostructure. The intense peak at approximately 8 keV originated from the copper grid.

Figure 6a shows a low-magnification TEM image of sample 1, which exhibits several nanostructures. Each individual nanostructure was capped with a clear spherical particle. EDX analyses of the particle and stem showed that this particle was composed mainly of Sn (69.4 at.%) and considerably small amounts of In (2.5 at.%) and O (28.1 at.%). Moreover, the stem of the nanostructure consisted mainly of In (44.4 at.%) and O (53.6 at.%) and a small amount of Sn (2.0 at.%). The analyses of the composition revealed that the O content of the stem was below the stoichiometric value of In_2_O_3_, which is consistent with the XPS O 1 *s* analysis. The presence of Sn-rich particles at the ends of the nanostructures indicated that the vapor–liquid-solid (VLS) process might be crucial for crystal growth. Several studies on the synthesis of In_2_O_3_ nanostructures have shown the importance of the Au catalytic layer for the formation of In_2_O_3_ nanostructures [23]. Most of the catalytic growth of oxide nanostructures through vapor transport follows a VLS crystal growth process [24]. In this work, no metallic thin layer was pre-deposited onto the substrates to act as a catalyst for nanostructure growth. Recently, a self-catalyst VLS growth mechanism was proposed to explain the growth of Mg-doped ZnO nanostructures and Zn-Sn-O nanowires [25,26]. The origin of the metallic Sn particles at the ends of our nanostructures might thus be similar to those of previously reported nanostructures. The selected TEM image taken from the corner of the particle-stem region of Figure 6b reveals a non-zero conical angle, demonstrating that the nanostructure geometry ended at a decreasing radius during growth (inset 1 in Figure 6b). The HRTEM image in Figure 6b shows clear lattice fringes corresponding to the (200) plane, which is perpendicular to the stem axis, of the cubic In_2_O_3_ structure. The sharp and bright spots in the selected area electron diffraction (SAED) pattern taken along the [001] zone axis show that the nanostructure was single crystalline and grew along the [100] axis (inset 3). Moreover, the SAED pattern of the particle could be indexed along the [010] zone axis of Sn (inset 4). The HRTEM image taken from the interface of particle and stem reveals a thin transition layer with a thickness of approximately 5 nm at the interface (inset 5). Below this transition layer, ordered lattice fringes of (200) for In_2_O_3_ were observed over the entire stem. The In and Sn element mapping images (Figure 6c) clearly show that In and Sn were uniformly distributed over the particle and stem of the nanostructure, respectively. These results demonstrate the compositional homogeneity of the as-synthesized nanostructure.

**Figure 7:**
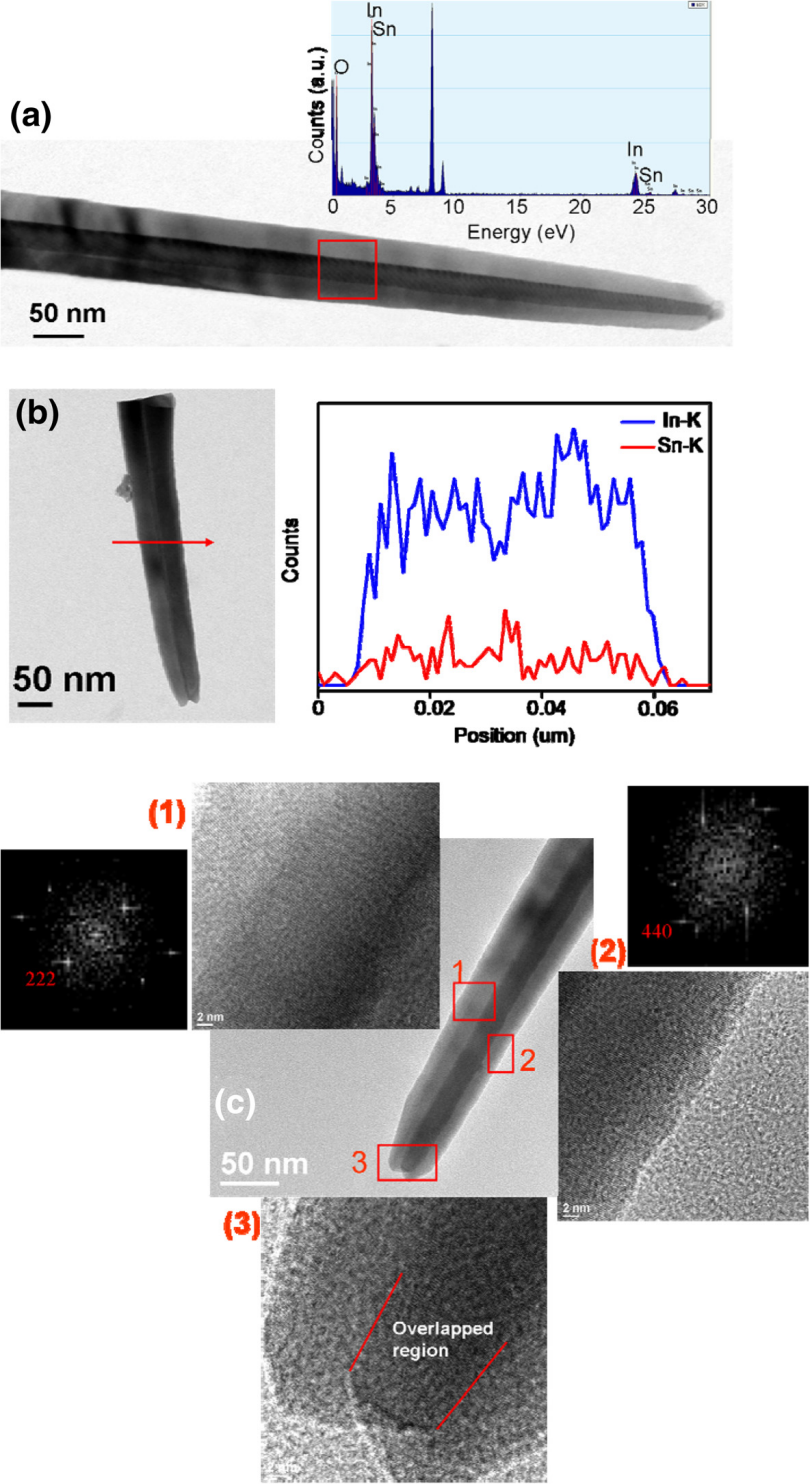
**TEM and HRTEM images of the sword-like nanostructures. (a)** Low-magnification TEM image and EDS spectrum of the single In-Sn-O nanostructure. **(b)** The low-magnification TEM image and the corresponding cross-sectional EDS line scan profiling of the sword-like nanostructure. **(c)** HRTEM images and corresponding FFT patterns taken from the various regions of the nanostructures. The intense peak at approximately 8 keV originated from the copper grid.

Figure 7a shows a low-magnification TEM image of the double-edged straight sword-like In-Sn-O nanostructure (sample 2). The nanostructure ends with a particle that has a diameter smaller than that of the stem. EDX analysis of the nanostructure shows that the stem consisted mainly of In and O, and the Sn content was approximately 2.4 at.% (inset in Figure 7a). Cross-sectional line scan profiling of the sword-like nanostructure showed that the major In and trace Sn elements were homogeneously distributed over the cross section of the stem (Figure 7b). Figure 7c shows the HRTEM images of individual sword-like nanostructures. The particle of the nanostructure disappeared during the preparation of the TEM sample. The HRTEM images were taken from different sides of the sword-like nanostructure. The corresponding fast Fourier transform (FFT) patterns demonstrated that the sword-like nanostructure was composed of two plates with different crystallographic orientations. Both high-resolution imaging and FFT patterns showed that the stems of the left (region 1) and right (region 2) plates mainly grew along the [111] and [110] directions, respectively. The high-magnification image of the tip region (region 3) of the nanostructure clearly revealed that parts of the two plates overlapped each other, resulting in a double-edged straight sword morphology.

**Figure 8:**
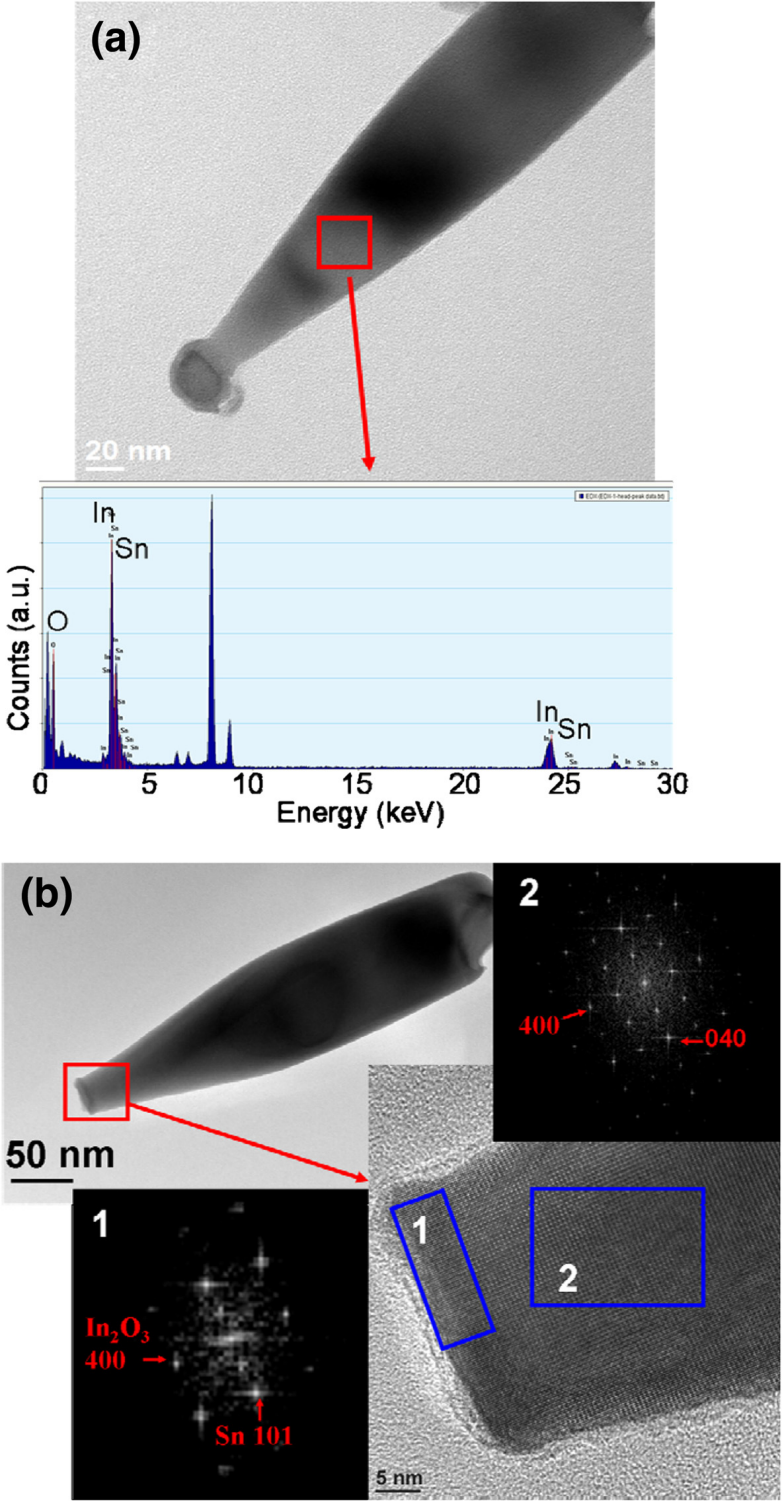
**TEM and HRTEM images of the bowling pin-like nanostructures. (a)** Low-magnification TEM image and EDS spectrum of the single In-Sn-O nanostructure. **(b)** HRTEM images and corresponding FFT patterns taken from the various regions of the nanostructures. The intense peak at approximately 8 keV originated from the copper grid.

Figure 8a shows a low-magnification TEM image of the bowling pin-like In-Sn-O nanostructure (sample 3). EDS analysis demonstrated that the stem of the nanostructure consisted mainly of In (40.8 at.%) and O (56.9 at.%), and a small amount of Sn (2.3 at.%). TEM images of the individual nanostructure without a particle (which disappeared during TEM sample preparation) were purposely selected to understand the interface status of the particle and stem (Figure 8b). The local HRTEM image and FFT patterns taken from the interfacial region and stem are shown in the insets of Figure 8b. According to the FFT pattern, the lattice fringes of the stem corresponded to the (200) plane of the cubic In_2_O_3_ structure, indicating that the nanostructure grew along the [100] direction. However, the interface region, which had a thickness of approximately 5 nm, showed lattice fringes that differed from those of the stem. The FFT pattern of the interface region clearly showed Sn spots that indicated that the thin interfacial layer was formed with a high metallic Sn content during crystal growth.

**Figure 9:**
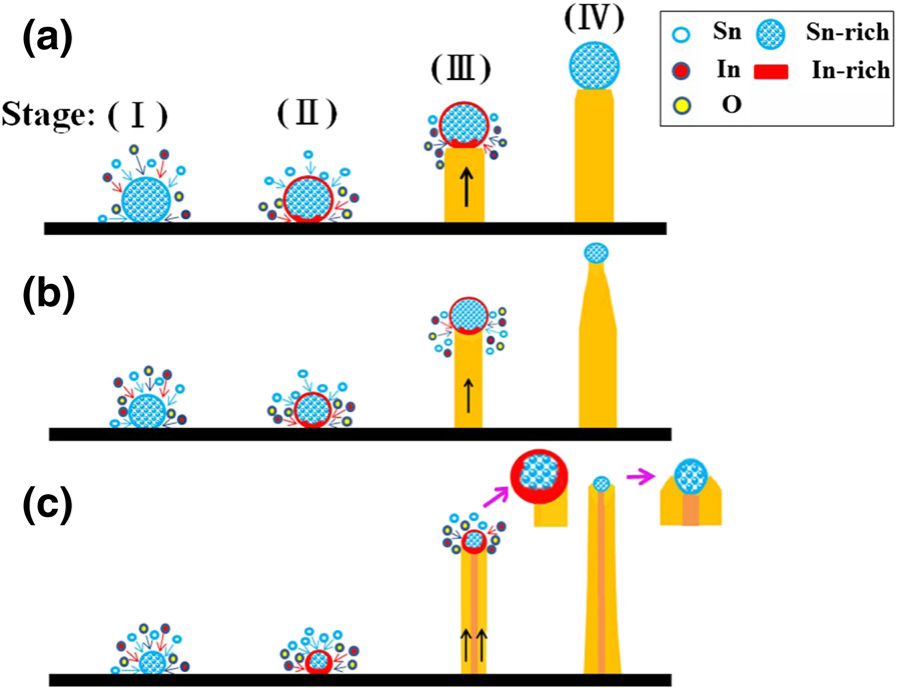
**Possible growth mechanisms of In-Sn-O nanostructures with various morphologies. (a)** The possible growth mechanism of the rod-like nanostructures. **(b)** The possible growth mechanism of the bowling pin-like nanostructures. **(c)** The possible growth mechanism of the sword-like nanostructures.

Figure 9 shows the possible growth mechanism of the nanostructures of various samples. The possible growth mechanism for sample 1 can be described as follows (Figure 9a). First, the evaporated Sn vapor forms Sn-rich (with trace In content) liquid droplets on the substrates (stage I). The low melting point (232°C) of Sn results in its re-vaporization and adsorption on the particle surface. If the Sn vapor concentration is sufficiently high, the adsorbed species that are transported from the vapor phase maintain the particle size during crystal growth. Because of further dissolution of the In and Sn vapors into the Sn-rich alloy droplets, In-rich alloys (with trace Sn content) are formed on the surface of the droplets. When more species transfer into the droplets, they become supersaturated, and most In with trace Sn (In-rich alloy) precipitates to the bottom of the droplets during growth (stage II). Simultaneously, the precipitated In-rich alloys oxidate at the bottom of the Sn-rich catalyst because of the residual oxygen in the furnace, and crystals grow along the direction perpendicular to the stem axis (stage III). Finally, the growth process leads to the formation of Sn-rich particles at the ends of the stems of the In-Sn-O nanostructures (stage IV). The nanostructures in sample 1 maintained their stem size during growth, and only a small segment of the stem near the terminal particle exhibited a decreased dimension because of the relatively low In vapor saturation toward the end of the experiment. Because nanostructure size depends on catalyst size within the framework of the VLS growth mechanism, the nanostructures in sample 1 may have grown predominantly through the VLS process. Comparatively, the particles in sample 1 had a considerably large diameter. The TEM images showed that the diameter of the particles in sample 1 was larger than 200 nm; however, those of sample 2 (approximately 15 nm) and sample 3 (approximately 30 nm) were relatively small. In the VLS growth mode, reaction temperature might affect the size of the catalyst particles at the tip of the nanostructures. Among the three samples, the position of sample 1 was the closest to the source materials in the reaction furnace. A high Sn vapor concentration tends to cause massive Sn atoms to agglomerate and form larger Sn-rich catalysts on the substrate; therefore, the large diameters of the nanostructures in sample 1 may have been produced through the VLS growth mechanism. The nanostructures in sample 3 exhibited a relatively large segment with a decreasing radius in the stem compared with that of sample 1. Therefore, stage II of the synthesis of the nanostructures of sample 3 might be different from that of the nanostructures in sample 1. The crystal growth (Figure 9b) of the bowling pin-like nanostructures in stage II is controlled through a VLS mechanism. However, a large segment with a decreasing radius might be indicative of a decreasing particle diameter during crystal growth. This may occur because the Sn species that are adsorbed from the vapor phase cannot maintain a stable particle size during crystal growth. At stage III, most of the adsorbed In and O species maintain 1D stem growth along the [100] crystalline direction because of sufficient In vapor saturation. By continuing the growth process, the saturation degree of the Sn vapor decreases constantly toward the end of the experiment. Finally, stems with a large segment exhibiting a decreasing radius and a terminal particle form (stage IV). The possible growth mechanism of the sword-like nanostructures in sample 2 is proposed as follows (Figure 9c). After Sn-rich alloy droplets form on the substrate (stage I), the major In-rich alloy forms under the supersaturated Sn-rich droplet, possibly with an extremely high concentration of In dissolved into the droplet (stage II). The spreading of In-rich alloys under the droplets results in the formation of nucleation sites for the growth of two In-rich alloy plates. Because the In vapor is sufficiently saturated around the substrate, the adsorbed species maintains the 1D growth of the two plates (stage III). In this stage, droplets are displaced from the center of the nanostructure axis of each plate (inset of stage III). Two In-rich alloy plates under the particles create a zero torque on the droplets, avoiding the particle shear off the nanostructure during crystal growth. Controlled by the VLS mechanism, the inner side of the plates overlaps each other because of the limitation of Sn-rich droplet size during the 1D crystal growth. Growth continues if In vapors keep dissolving into the droplet, and, finally, a double-side sword-like nanostructure forms (stage IV).

**Figure 10:**
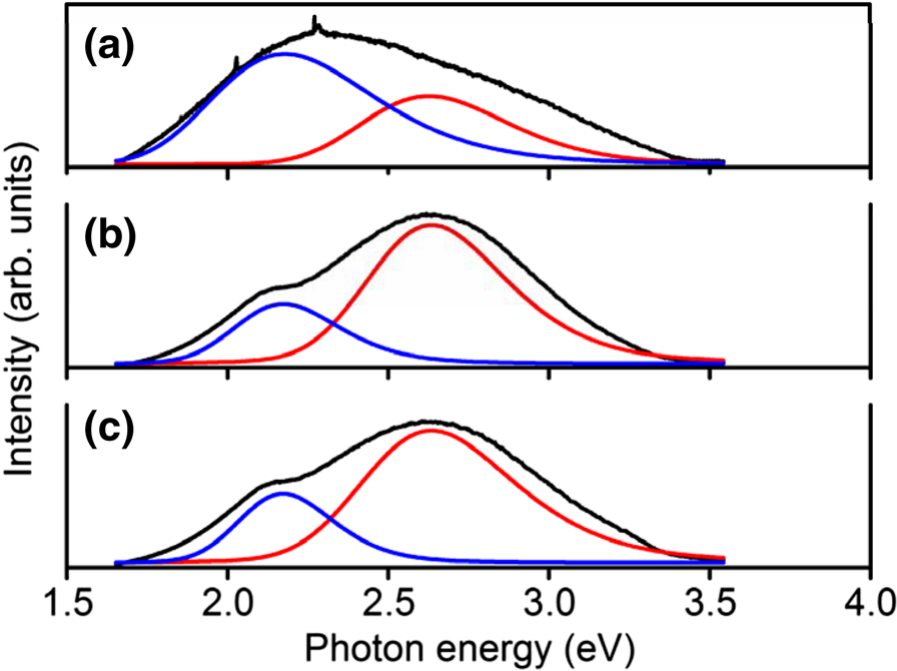
PL spectra of In-Sn-O nanostructures: (a) sample 1, (b) sample 2, and (c) sample 3.

The PL spectra of the In-Sn-O nanostructures at room temperature were analyzed (Figure 10). Broad visible emission peaks were observed. These peaks were fitted by two Gaussian-resolved peaks centered at approximately 2.17 and 2.63 eV, which correspond to the yellow-orange and blue-green emission bands, respectively. Several studies have reported the deep level emissions of In_2_O_3_ nanostructures. However, the origin of the deep level emission band remains unclear. Oxygen vacancies near the surface of the In_2_O_3_ nanostructures are associated with yellow-orange emissions [24,27]. By contrast, oxygen vacancies have been attributed to the green emission band [28]. XPS and TEM-EDS analyses indicated that the Sn content of the nanostructures of sample 1 (2.0 at.%) was slightly lower than those of sample 2 (2.4 at.%) and sample 3 (2.3 at.%). Moreover, the density of oxygen vacancies at the surface of the nanostructures was relatively high in sample 1 (39%) compared with those in sample 2 (28%) and sample 3 (21%). Comparatively, the ratio of yellow-orange emission band to total visible emission band for sample 1 (72.2%) was larger than those of sample 2 (32.3%) and sample 3 (32.0%). Our results suggested that the oxygen vacancies near the surface of the nanostructures might dominate the yellow-orange emission band. Recent work on the PL spectra of In-Sn-O nanostructures has shown that a relatively high Sn content (3.8 at.%) in the nanostructures causes a clear blueshift (590 to 430 nm) in the visible emission band [15]. Kar et al. reported that the blue-green emission band of In_2_O_3_ can be attributed to oxygen vacancies and indium-oxygen complex vacancy centers, in which indium-oxygen vacancy centers may act as the acceptors after excitation [29]. The blue-green emission bands in this study might be associated with the recombination of electrons from Sn doping, which induced a new defect level through photoexcited holes [15,29].

## Conclusions

In conclusion, crystalline In-Sn-O nanostructures with three morphologies (rod-like, sword-like, and bowling pin-like) were obtained through thermal evaporation using mixed metallic In and Sn powders. The nanostructures were capped with Sn-rich particles of various sizes. Nanostructure formation was achieved through self-catalytic growth. Sn-rich alloy particles promoted the formation of In-Sn-O nanostructures during thermal evaporation. Sn vapor saturation around the substrate played a key role in determining the size of the Sn-rich alloy droplets and thus affected the final morphology of the 1D nanostructures. Detailed composition and elemental binding energy analyses showed that the PL properties of the In-Sn-O nanostructures consisted of blue-green and yellow-orange emission bands and were associated with the Sn content and crystal defects of the nanostructures.

## Competing interests

The authors declare that they have no competing interests.

## Authors' contributions

Y-CL designed the experiments, carried out the material analyses, and drafted the manuscript. HZ carried out the sample preparations. Both authors read and approved the final manuscript.
